# Matrix Metalloproteinase 2 Is Required for Ovulation and Corpus Luteum Formation in *Drosophila*

**DOI:** 10.1371/journal.pgen.1004989

**Published:** 2015-02-19

**Authors:** Lylah D. Deady, Wei Shen, Sarah A. Mosure, Allan C. Spradling, Jianjun Sun

**Affiliations:** 1 Department of Physiology & Neurobiology, University of Connecticut, Storrs, Storrs, Connecticut, United States of America; 2 Howard Hughes Medical Institute Research Laboratories, Department of Embryology, Carnegie Institution for Science, Baltimore, Maryland, United States of America; 3 Institute for Systems Genomics, University of Connecticut, Storrs, Storrs, Connecticut, United States of America; The University of North Carolina at Chapel Hill, United States of America

## Abstract

Ovulation is critical for successful reproduction and correlates with ovarian cancer risk, yet genetic studies of ovulation have been limited. It has long been thought that the mechanism controlling ovulation is highly divergent due to speciation and fast evolution. Using genetic tools available in *Drosophila*, we now report that ovulation in *Drosophila* strongly resembles mammalian ovulation at both the cellular and molecular levels. Just one of up to 32 mature follicles per ovary pair loses posterior follicle cells (“trimming”) and protrudes into the oviduct, showing that a selection process prefigures ovulation. Follicle cells that remain after egg release form a “corpus luteum (CL)” at the end of the ovariole, develop yellowish pigmentation, and express genes encoding steroid hormone biosynthetic enzymes that are required for full fertility. Finally, matrix metalloproteinase 2 (Mmp2), a type of protease thought to facilitate mammalian ovulation, is expressed in mature follicle and CL cells. Mmp2 activity is genetically required for trimming, ovulation and CL formation. Our studies provide new insights into the regulation of *Drosophila* ovulation and establish *Drosophila* as a model for genetically investigating ovulation in diverse organisms, including mammals.

## Author Summary

Sexual reproduction is thought to be a highly divergent process due to fast evolution and speciation. For example, sperm from one species can seldom fertilize eggs from another species, indicating that different molecular machinery for fertilization is applied in different species. In contrast to this divergent view, ovulation, the process of liberating mature eggs from the ovary, is a general phenomenon throughout the Metazoa. We provide evidence that basic mechanisms of ovulation are conserved. Like mammalian follicles, *Drosophila* follicles consist of single oocytes surrounded by a layer of follicle cells. *Drosophila* follicles degrade their posterior follicle cells to allow the oocyte to rupture into the oviduct during ovulation. The residual postovulatory follicles reside in the ovary, accumulate yellowish pigmentation, and produce the steroid hormone ecdysone, features which resemble the mammalian corpus luteum. We also showed that matrix metalloproteinase, a type of proteinase proposed to degrade the mammalian follicle wall during ovulation, is required in *Drosophila* for posterior follicle cell degradation and ovulation. These findings are particularly important because this simple genetic model system will speed up the identification of many conserved regulators required for regulating matrix metalloproteinase activity and ovulation in human, processes that influence ovarian cancer formation and cancer metastasis.

## Introduction

Ovulation, the liberation of a mature oocyte from the ovary, is one of the critical events of metazoan reproduction. In mammals, where ovulation has been studied most thoroughly, several important steps have been identified [1–4]. First, among a cohort of mature ovarian follicles, a dominant follicle arises. Eventually, proteolytic enzymes are locally activated that digest a small part of the dominant follicle’s wall and extracellular matrix, releasing the oocyte into the oviduct [5]. Finally, residual follicular cells remodel the ruptured follicle into the yellowish corpus luteum, an endocrine body that secretes the steroid hormones progesterone, estrogen, and other factors. While much has been learned, genetically testing the roles proposed for specific genes and pathways has been difficult. For example, the importance of matrix metalloproteinases (Mmps) in ovulation has not been demonstrated using knockout mice, possibly due to redundancy [6–9]. A genetically tractable system containing fewer redundant genes such as *Drosophila* would greatly facilitate ovulation studies. However, ovulation in *Drosophila* has not been well characterized and is not known to involve the same processes as mammalian ovulation.

**Fig 1:**
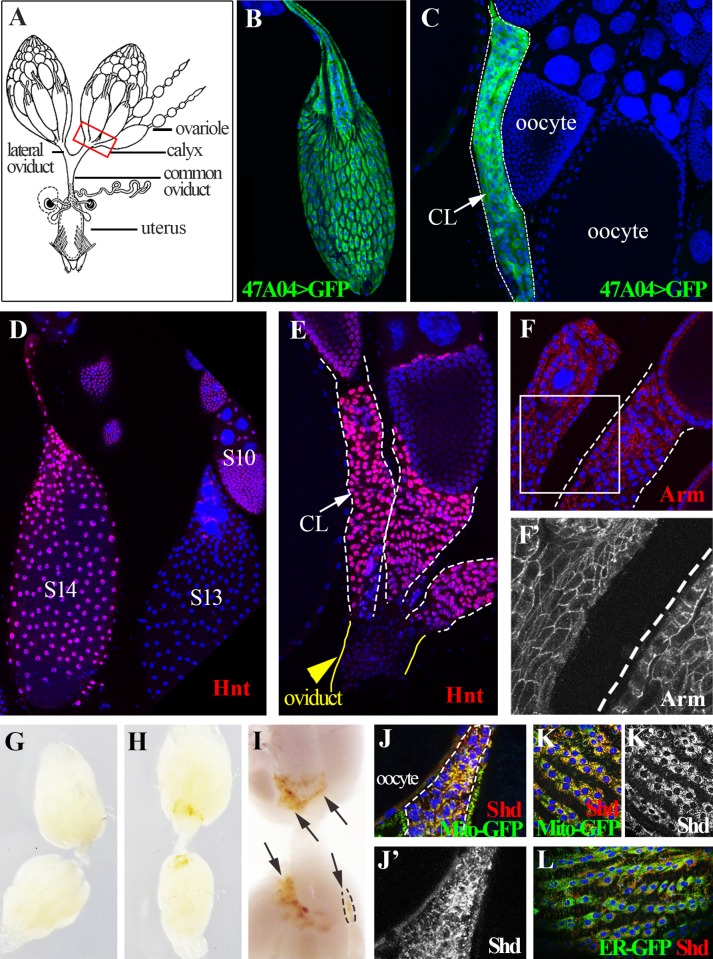
*Drosophila* follicle cells remain in the ovary following ovulation and form a corpus luteum. (A) A schematic diagram of female reproductive system. The red squared area is the calyx where post-ovulatory follicle cells are located. (B) R47A04-Gal4 driving UAS-GFP expression (R47A04>GFP) is specifically in follicle cells of S14 egg chambers (mature follicles). (C) Follicle cells from post-ovulatory follicles (green; outlined) remain in the ovary after the egg enters the oviduct and form a corpus luteum (CL). (D) Hnt accumulates in S14 follicle cells after decreasing in S13. (E) Hnt expression continues in CL cells. (F and F’) CL cells continue to express Arm, an adherens junction component (Red in F, white in F’) suggesting that they maintain their apical-basal polarity. G) Ovaries of virgin females before egg laying. No pigmentation is found in the ovary. (H, I) Ovaries of mated females after egg laying. Yellow pigmentation (arrows) is found in the CL. A single CL is outlined. (J-L) Shade (Shd) is expressed in CL cells (dash-line outlined in J and J’) and S14 follicle cells (K, K’, and L). Shd expression co-localized with a mitochondria marker (yellow in J and K), but not with an endoplasmic reticulum marker (L).

The *Drosophila* female reproductive system is anatomically similar to mammals, having two ovaries connected by lateral and common oviducts to the uterus, where fertilization occurs and one egg is retained prior to laying (Fig. 1A) [10]. Ovulation does not follow a simple cycle, however. Multiple eggs are laid when suitable food resources are available [11], and ovulation follows each oviposition to replenish the uterus. Egg laying and ovulation are extensively regulated by octopaminergic neural inputs [12–14] and can be elicited by peptides transferred in semen from the male [15–18]. Ovulation requires reproductive tract secretions controlled by the NR5a class nuclear hormone receptor Hr39 [19]. A mammalian ortholog, LRH-1, is required in mouse granulosa cells for ovulation, to maintain progesterone production in the corpus luteum and for decidual cell function in the uterus [20,21]. These similarities highlight the potential value of *Drosophila* as a genetically tractable model of ovulation.

Here, we show that a similar follicle rupturing process leads to *Drosophila* ovulation. Posterior follicle cells of a mature egg chamber are first degraded and the residual follicle cells are squeezed toward the anterior while the oocyte moves posteriorly into the lateral oviduct. Membrane-tethered Mmp2, but not Mmp1, functions in follicle cells to control follicle rupture and oocyte release. Residual follicle cells remain in the ovary, accumulate yellowish pigmentation, express ecdysone biosynthetic genes, and persist for an extended period before degradation, events reminiscent of the corpus luteum in mammals. Our data indicate that the cellular and molecular regulation of ovulation has been more conserved than previously thought.

## Results

### Follicles rupture to release the oocyte and form a corpus luteum

The fate of *Drosophila* follicle cells after ovulation has not been clearly described. If ovulation involves a programmed rupture of the follicular layer as in mammals, then most follicle cells would remain behind in the ovary after the egg is released into the oviduct. Alternatively, follicle cells might degenerate randomly or some might accompany the oocyte into the oviduct and the uterus. Using a mature (stage 14) follicle cell marker (Fig. 1B, Methods), we observed a cluster of GFP-labeled cells at the posterior end of each ovariole in the basal (calyx) region of ovary (Fig. 1C). In contrast, few, if any, follicle cells leave the ovary upon ovulation because GFP-labeled cells were not seen associated with eggs in the oviduct or uterus.

Post-ovulation mammalian follicles transform into the corpus luteum and similar behavior was reported previously in several other insects [22]. Consequently, we termed each cluster of residual Drosophila ovarian follicle cells as a corpus luteum (CL), and observed that CL cells continue a program of gene expression. The zinc-finger transcription factor Hindsight (Hnt), a major follicle cell regulator [23], is upregulated in stage 14 follicle cells and is expressed in CL cells (Fig. 1D-E). Expression of the adherens junction protein Arm (Fig. 1F-F’), suggests that CL cells maintain apical-basal epithelial polarity. Most dramatically, CL cells acquire a yellowish pigmentation (Fig. 1G-I) and express the ecdysone biosynthetic enzymes [24,25] Shade (Shd) in mitochondria (Fig. 1J-L) and Phantom (Phm) in the endoplasmic reticulum (ER; S1A Fig.). To examine the functional significance of steroid biosynthetic gene expression, we knocked down expression of *shd* and *phm*, *as well as dib* (*disembodied*, encoding another enzyme in the ecdysone synthesis pathway), in CL and mature follicle cells. These females all laid significantly fewer eggs than control females (S1B Fig.). This functional requirement for steroid biosynthetic enzymes supports the view that the *Drosophila* CL and mature follicle cells produces a steroid hormone such as ecdysone or 20-hydroxyecdysone, consistent with a recent finding that follicle cells in middle stages of oogenesis produce ecdysone to regulate border cell migration [26] and also reminiscent of steroid hormone production by the mammalian corpus luteum. We observed that only one CL is present in each ovariole, hence each CL must either degrade *in situ* over time following ovulation, or be extruded from the ovariole when the next egg is ovulated.

**Fig 2:**
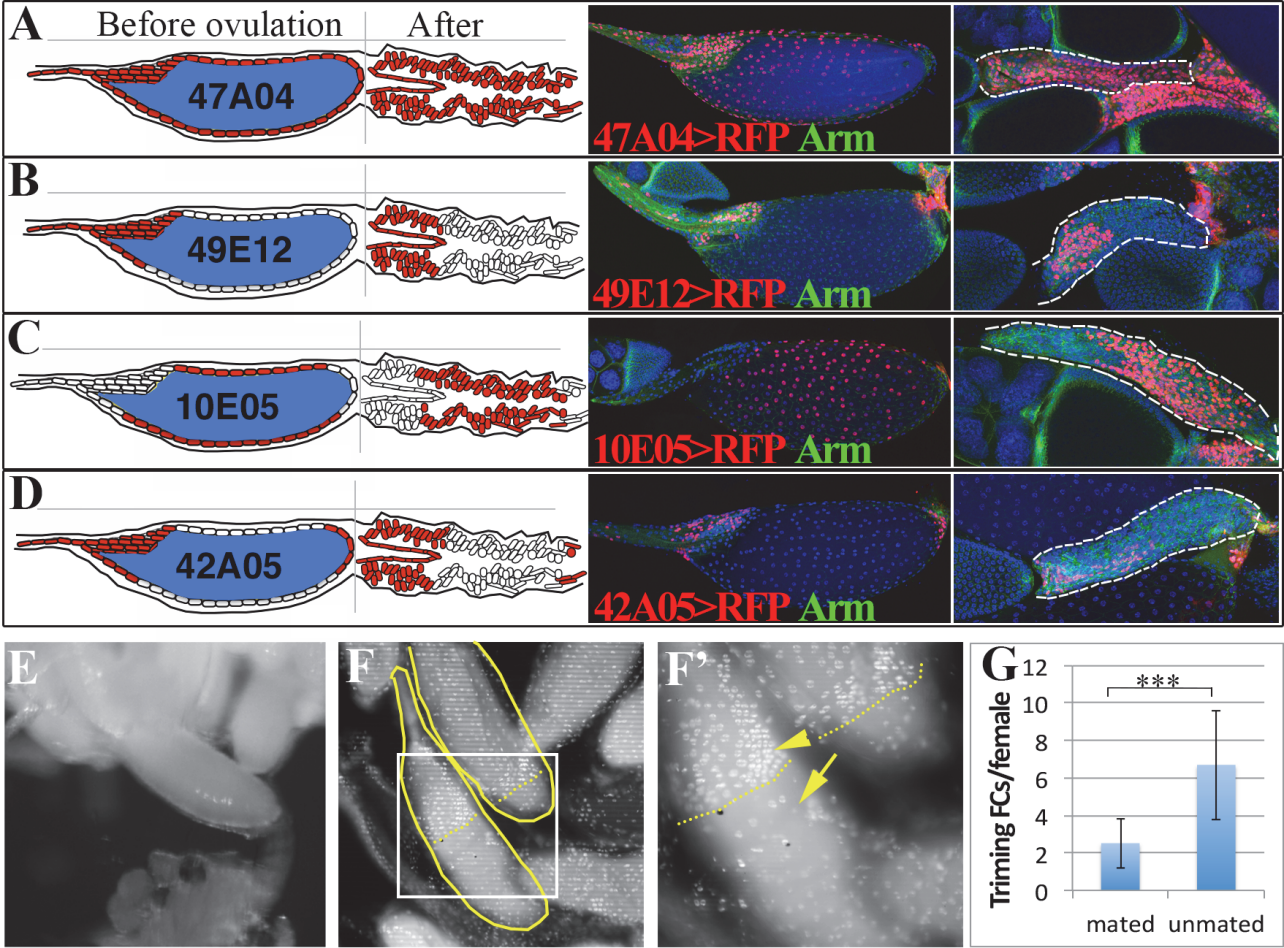
Posterior follicle cells are removed to release the oocyte and initiate corpus luteum formation. (A-D) Schematic (left panels) and real (right panels) expression patterns of Gal4 drivers in follicle cells of mature egg chambers and corpus luteum. Anterior follicle cells (B and D) reside at the anterior part of the corpus luteum, the middle follicle cells (C) reside at the middle portion of the corpus luteum, while the posterior follicle cells (D) are degraded in the corpus luteum. (E) An egg partially extruded into one lateral oviduct. (F-F’) DAPI stained calyx region showing the presence of two stage 14 trimming follicles (outlined) with no follicle cells at the posterior ends. The one with most posterior follicle cells trimmed was half-way in the lateral oviduct. The posterior end of follicle cells (brighter and larger nuclei pointed with an arrowhead) was marked by dashed lines, and the oviduct cells (faint and smaller nuclei) was marked by an arrow. F’ is the higher magnification of the squared region in F. (G) Histogram showing the number of follicles undergone trimming in mated (optimal ovulation) and unmated (no ovulation) females. Student’s T-test is used (*** P<0.001).

The organization of the CL reflects its origin in the follicle. All CL cells are labeled by a mature follicle cell driver, suggesting that cells from other sources are absent (Fig. 2A). Little cellular rearrangement occurs, since only anterior or middle cells of the corpus luteum were labeled by lines specifically expressed in anterior or middle stage 14 follicle cells (Fig. 2B-C). Lines specifically expressed in the posterior follicle cells did not label the CL, suggesting that these cells were degraded during ovulation (Fig. 2D).

### Trimming of posterior follicle cells and protrusion precedes ovulation

*Drosophila* ovaries each usually contain at least 15 mature follicles, one per ovariole, oriented with their posterior ends facing the oviduct, raising the question of how one particular follicle is selected for ovulation. We examined ovary pairs from females cultured under conditions favorable for egg laying and found that at most one mature follicle protrudes significantly into a lateral oviduct (Fig. 2E). The protruding follicle always lacked posterior follicle cells covering the part of egg inside the lateral oviduct (Fig. 2F). We termed this process of losing posterior follicle cells as “trimming”. The trimmed follicle’s location indicates that trimming and protrusion represent preludes to ovulation. Frequently, another stage 14 follicle was present that had lost a smaller area of posterior follicle cells, but did not protrude (Fig. 2F-G and S2A-C Fig.), which likely represents the next follicle to ovulate. These observations show that a follicle is preselected in *Drosophila* well before ovulation, undergoes trimming, and awaits the next ovulation event while protruding into the lateral oviduct. In flies that were laying few eggs, for example in unmated females, up to 6 trimmed follicles could be present per female (Fig. 2G), but the follicle with the greatest level of trimming continues to protrude into the oviduct and remains in a position poised for ovulation.

### Gelatinase activity is associated with trimming and egg release

**Fig 3:**
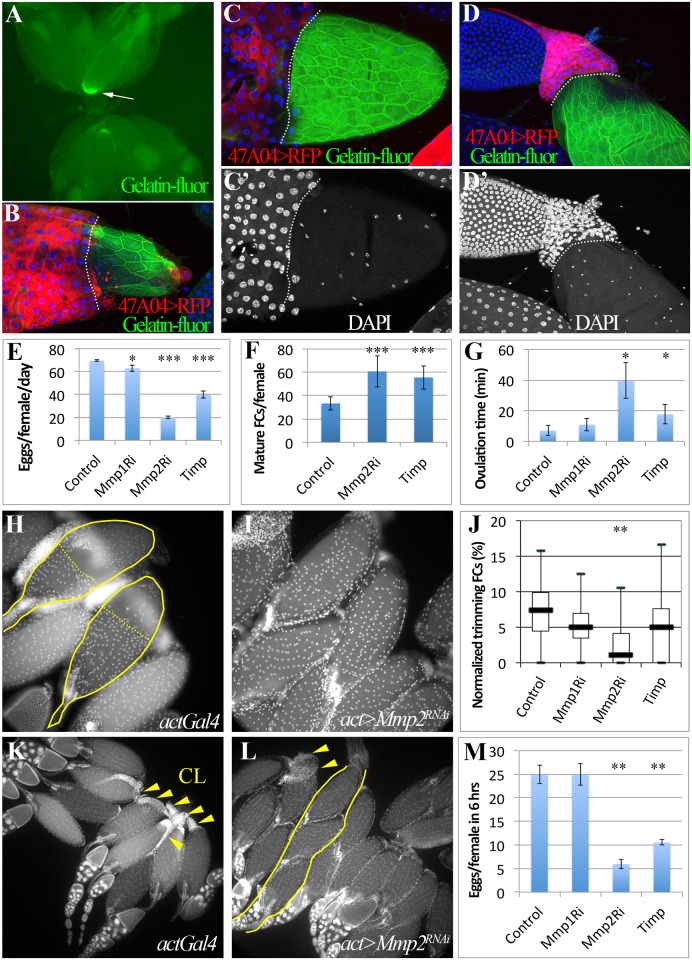
Mmp2 is required for ovulation and CL formation. (A) In situ zymography showing one preselected follicle with high gelatinase activity at its posterior end in the entire ovary pair. (B-D) Correlation of follicle cell trimming and gelatinase activity (green in B-D). During early (B), middle (C-C’), and late (D-D’) ovulation, gelatinase activity is covering all the egg chamber area lost follicle cells. Posterior leading edge of the follicle cell layer was marked by dashed lines. Smaller nuclei (white in C’ and D’) are oviduct cells. (E-G) Egg laying (E), mature follicles in ovary (F), and the average ovulation time (G) of females of *actGal4* or *actGal4* driven *Mmp1* ^*RNAi*^, *Mmp2* ^*RNAi*^, or *Timp* expression in adult. (H-I) Follicle cell trimming in actGal4 control (H) and actGal4/Mmp2RNAi (I) ovaries. Mature egg chambers with posterior follicle cell trimmed were outlined with solid line and the posterior edge of the residual follicle cells were marked with dashed line. Follicle cell nuclei are elucidated by DAPI signal. (J) A quantification of trimming follicles in ovaries. (K-L) More corpora lutea (arrowheads) are found in actGal4 control ovaries (K) than those in actGal4/*Mmp2* ^*RNAi*^ ovaries (L) 6 hours after mating. One ovariole with three mature egg chambers was outlined in (K). (M) Number of eggs laid in 6 hours after mating. T-test is used (*** P<0.001, ** P<0.01, * P<0.05).

The study of explanted mammalian follicles strongly implicates matrix metalloproteinases (Mmps) as important contributors to oocyte release [5]. In these follicles, Mmp activity is localized to the apex region where rupture will later occur [6]. We carried out gelatinase assays *in situ* to measure localized Mmp activity within *Drosophila* follicles before and during ovulation. High localized activity was found at the posterior end of one mature follicle in each ovary pair while a second follicle sometimes had lesser activity (Fig. 3A); the location of the activity correlated with the site of follicle cell trimming at the posterior (Fig. 3B). As eggs begin to enter the oviduct, the fraction of the follicular surface with gelatinase activity (Fig. 3C) increased from posterior to anterior, and matched where follicle cells no longer reside (Fig. 3C’). In eggs that have nearly separated from their follicle cells, gelatinase activity covered the entire surface (Fig. 3D), however, the anterior and middle follicle cells remained in a mass at the base of the ovary (Fig. 3D’). These data tightly associate Mmp activity with posterior follicle cell trimming, and suggest that more anterior Mmp activity subsequently degrades just the extracellular matrix separating the oocyte from intact middle and anterior follicle cells.

### Mmp2 is genetically required for ovulation and CL formation

*Drosophila* has two genes encoding matrix metalloproteinases, *mmp1* and *mmp2*, and one *Timp (Tissue inhibitor of matrix metalloproteinase*)[27]. Genetically reducing Mmp2 but not Mmp1 expression dramatically lowered egg laying (Fig. 3E; S3 Fig.). Mature egg chambers accumulated in Mmp2 knockdown females (Fig. 3F), indicating a block in ovulation, and the average time required to ovulate (see Methods) increased fivefold (Fig. 3G and Table 1). Similarly, overexpressing Timp, a protein that inhibits both Mmp1 and Mmp2 activity, also decreased egg laying and increased egg retention and ovulation time (Fig. 3E-G, Table 1). These data show that Mmp2 enzymatic activity is required for normal ovulation in *Drosophila*.

**Table 1:** The effect of Mmp activity on egg laying, egg distribution in reproductive tract, and egg laying time.

Genotype	Egg laying in 2 days^#^		Egg distribution in 6h		Egg laying time (min)		
	N	Eggs/ female/ day	N	Uterus with egg (%)	Total time	Ovulation time	Uterus time
*actGal4/+; tubGal80^ts^/+*	45	69.5 ± 0.5	30	63.3 ± 17.3	19.0 ± 0.1	7.0 ± 3.3	12.0 ± 3.3
*actGal4/+; tubGal80^ts^/UAS-mmp1^RNAi^*	25	62.9 ± 2.3*	27	48.1 ± 18.9	21.0 ± 0.8*	10.9 ± 4.0	10.1 ± 4.0
*actGal4/+; tubGal80^ts^/UAS-mmp2^RNAi^*	40	20.0 ± 0.8***	30	40.0 ± 17.5	66.1 ± 2.7*	39.7 ± 11.7*	26.5 ± 11.6*
*actGal4/+; tubGal80ts/UAS-Timp*	25	40.1 ± 2.8***	28	46.4 ± 18.5	32.9 ± 2.3*	17.6 ± 6.2*	15.3 ± 6.2
*UAS-dcr2/+; 47A04Gal4/+*	50	64.1 ± 1.8	24	41.7 ± 19.7	20.6 ± 0.6	12.0 ± 4.1	8.6 ± 4.1
*UAS-dcr2/+; 47A04Gal4/UAS-mmp2^RNAi^*	25	36.3 ± 3.8***	32	37.5 ± 16.8	36.3 ± 3.3*	22.7 ± 6.4*	13.6 ± 6.2
*UAS-dcr2/+; 47A04Gal4/UAS-Timp*	25	40.5 ± 1.5***	25	28 ± 17.6	32.6 ± 1.0*	23.5 ± 5.8*	9.1 ± 5.7
*42A05Gal4/UAS-mmp2^RNAi^*	20	45.7 ± 3.5**					

# one day = 22h at 29°C

* P<0.05,

** P<0.01,

*** P<0.001

All data are mean ± 95% confidence interval. Ttest was used for egg laying, while Fisher’s exact test was used for egg distribution.

Mmp2 was also required for follicle trimming (Fig. 3H-I and Table 2). The rate of trimming was reduced at least three fold in Mmp2 knock down animals both before mating and at 6-hour post mating (Fig. 3J and Table 2). In addition, Mmp2 knock down ovaries lacked corpus luteum structures (Fig. 3K-L), and instead accumulated mature egg chambers (Fig. 3L). Both Mmp2 knock down females and females expressing Timp, displayed severe egg laying defects even within 6 hours of mating (Fig. 3M). Thus, Mmp2 activity is required in adult females for follicle cell trimming, ovulation, corpus luteum formation, and egg laying.

**Table 2:** The effect of Mmp activity for follicle cell trimming.

	6 hour post mating				Unmated virgin female			
Genotype	No. of females	Mature eggs/female	Posterior trimmed eggs/female	Normalized trimming eggs/ female (%)	No. of females	Mature eggs/female	Posterior trimmed eggs/female	Normalized trimming eggs/ female (%)
*actGal4/+; tubGal80^ts^/+*	14	21.6 ± 5.8	1.5 ± 0.9	7.2 ± 5.2	19	22.1 ± 10.2	2.6 ± 1.2	12.4 ± 4.7
*actGal4/+; tubGal80^ts^/UAS-mmp1^RNAi^*	18	36.1 ± 9.8 ***	1.8 ± 0.9	5.4 ± 3.2				
*actGal4/+; tubGal80^ts^/UAS-mmp2^RNAi^*	24	31.3 ± 8.3 ***	0.8 ± 0.9 *	2.5 ± 3.2 **	14	37.2 ± 13.6 ***	0.1 ± 0.4 ***	0.5 ± 1.6 ***
*actGal4/+; tubGal80ts/UAS-Timp*	20	34.7 ± 8.8 ***	1.8 ± 1.6	5.3 ± 5.1	17	48.0 ± 13.1 ***	3.9 ± 3.4	7.6 ± 5.8 **

* P<0.05,

**P<0.01,

***P<0.001

All data are mean ± SD. Ttest was used.

### Mmp2 is expressed and functions in follicle cells to control ovulation

**Fig 4:**
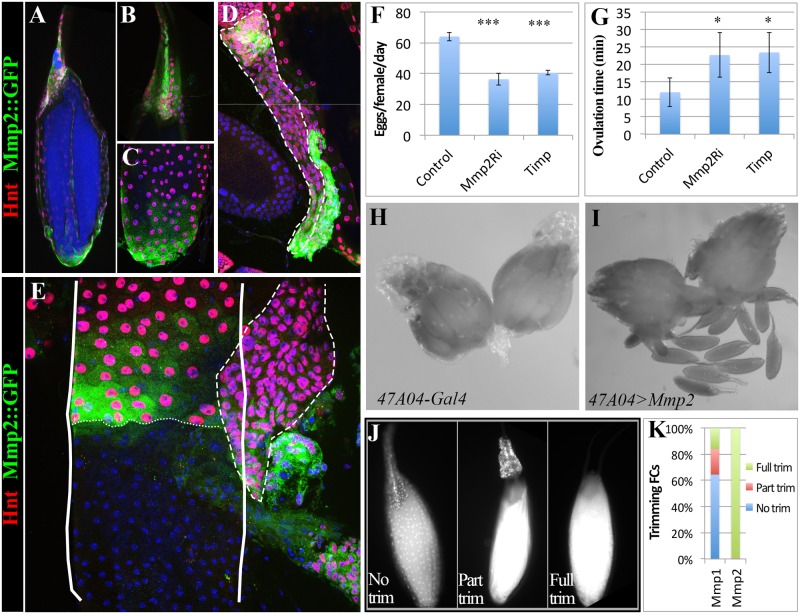
Mmp2 functions in follicle cells to control ovulation. (A-C) Mmp2 expression in mature egg chambers indicated by Mmp2::GFP fusion protein. Mmp2 is highly expressed in both anterior (B) and posterior (C) follicle cells. Hnt (Red) is used to mark follicle cells. (D) Mmp2::GFP is expressed in anterior and posterior corpus luteum (outlined with dashed line). (E) Mmp2::GFP is expressed in the posterior edge of the trimming follicle cells when the egg is half way in the oviduct. The egg chamber is outlined by solid line, the posterior edge of follicle cells is marked by small dashed lines, and the corpus luteum is outlined by big dashed line. Smaller nuclei without Hnt expression are from oviduct epithelial cells. (F-G) The egg laying (F) and ovulation time (G) of females with *47A04-Gal4* or *47A04-Gal4* driven *Mmp2* ^*RNAi*^ or *Timp* expression in mature follicle cells. T-test is used (*** P<0.001, *P<0.05). (H-I) Ectopic Mmp2 in mature follicle cells is sufficient to cause egg release from ovary into abdominal cavity. (J) DAPI staining to indicate the follicle cell trimming of egg chambers released from ovary ectopic Mmp2 expression in (I). (K) Quantification of egg chamber trimming. Egg chambers already released from ovaries were collected from abdominal cavity of females with 47A04 driven Mmp1 or Mmp2 expression. 149 and 144 released egg chambers were collected from Mmp1 and Mmp2 flies respectively.

We generated an in vivo Mmp2::GFP fusion allele at its normal genomic location by swapping an in-frame GFP exon into a MiMIC transposon inserted within an *Mmp2* coding intron (S4 Fig.). We also employed a Gal4 enhancer trap line (see Methods), which mimics Mmp2 expression during pupal imaginal disc eversion, to monitor Mmp2 transcription. Mmp2 fusion protein and RNA are specifically expressed in posterior follicle cells in all mature stage 14 follicles but not in earlier follicles (Fig. 4A-C, S4C Fig. and S5A-D Fig.). Mmp2 is also expressed in some anterior follicle cells that help form dorsal eggshell structures. The reporters show expression at the posterior edge of surviving follicle cells during trimming, and in anterior and posterior corpus luteum cells (Fig. 4D-E and S5D Fig.).

We interfered with Mmp2 expression specifically in mature follicle cells by using a mature follicle cell driver (R47A04) to express Mmp2 RNAi or to overexpress Timp, and observed that ovulation and egg laying were defective (Fig. 4F-G and Table 1). The defect is not likely due to disruption of Mmp2 in neurons as R47A04 is not expressed in sensory neurons innervating the female reproductive tract (S5E-F Fig.). Action outside of neurons is also supported by the observation that knocking down Mmp2 with a more restricted mature follicle cell driver (R42A05: expressed in posterior and anterior mature follicle cells; Fig. 2D) showed a similar egg laying defect (Table 1), although one of lower severity. When Mmp2 was overexpressed in mature follicle cells, mature eggs ruptured and were released into the female abdominal cavity (Fig. 4H-I). Most such eggs lacked covering follicle cells (Fig. 4J-K). When Mmp1 was ectopically expressed in mature follicle cells with the same Gal4 driver, fewer follicles ruptured into the abdominal cavity and most released eggs retained some follicle cells (Fig. 4K). Consequently, Mmp2 is required in mature follicle cells to trim the follicular layer leading to ovulation, and its level of expression must be controlled or normal activity regulation may be overwhelmed.

## Discussion

Despite different biological strategies of ovulation in mice and *Drosophila*, our studies reveal strong similarities in the underlying mechanisms. In both species, a follicle is selected before ovulation, and its oocyte is released at an appropriate time by inducing Mmp proteolytic activity, either in the apex region (mouse) or at the follicle posterior (*Drosophila*). Mmp2 activation is likely controlled by pro-domain processing, and may also be modulated at the level of protein secretion and/or by the presence of the endogenous inhibitor Timp. Pharmacological inhibition of Mmp activity prevents ovulation in vitro in mice [28,29], as well as in other vertebrate and primate species [30,31]. However, knockouts of individual Mmp genes have not been reported to affect ovulation, presumably due to redundancy, although individual Mmp gene knockouts frequently have specific phenotypic effects (reviewed in [9]). In contrast, our studies clearly show a genetic requirement of Mmp2 but not Mmp1 for trimming, ovulation and CL formation. However, follicles from MMP2 RNAi females still retained some gelatinase activity based on the in situ assay, and our experiments cannot rule out that MMP1 or other proteases also contribute to follicle trimming.

The value of *Drosophila* for studies of ovulation is further illustrated by the discovery that after ovulation, residual follicle cells form a corpus luteum. The corpus luteum (Latin for “yellow body”) was first described by Volcherus Coiter in 1573, but its relationship to ovulation rather than pregnancy was not understood until the early 19^th^ century [32]. The existence of a pigmented structure in insect ovaries was also recognized in the 19^th^ century, at least in a few species [22]. However, it has remained unclear whether a CL exists in *Drosophila*, whether it is a universal feature of insect oogenesis, whether the CL functions in reproduction, and whether any such functions have been conserved during evolution. Our studies indicate that a CL is formed in *Drosophila* and that Mmp2 activity is required for its production. The mammalian CL contains at least two cell types, small CL cells which are thought to be derived from thecal cells, and large CL cells that produce progesterone. Our gene expression studies suggest that at least two cell types are also likely in the *Drosophila* CL [33]. Some anterior CL cells may derive from stretch cells, which never secrete eggshell proteins [34].

The *Drosophila* CL may function at least in part by producing the steroid hormones, ecdysone or 20-hydroxyecdysone, as suggested by continuous expression of genes encoding P450 enzymes Phantom and Shade in CL cells. Mated females are known to have a higher ecdysone titer than unmated females [35], consistent with the idea that the CL contributes substantially to ecdysone production. A common role in steroid hormone production might explain the conserved pigmentation of the CL. In mammals, carotenoid accumulation is beneficial to gametogenesis and is associated with steroid hormone production. These molecules may influence free radical balance, which might otherwise interfere with steroid production [36]. Alternatively, carotenoids may simply accumulate because they are found within the circulating lipoprotein particles that must be taken up to support steroid production [37]. The easy of genetic manipulation in *Drosophila* may allow the biochemical nature and function of the yellow pigmentation in the CL to be further characterized.

Finally, we propose that a major function of the CL in *Drosophila* is to control the maturation of younger follicles in the ovariole, and to select mature follicles for the next ovulation. In their location at the posterior end of each ovariole, CL cells are well positioned to govern the orderly usage of mature follicles. If CL cell secretory activity decreases with age, the corpus luteum in each ovariole might communicate the elapsed time since an ovariole was last used, for example by local inhibition, promoting the relatively uniform usage of all ovarioles. Although the large scale organization of follicles within the mammalian ovary is less obvious, signals from its corpora lutea might likewise spatially control follicle maturation. Knowledge that some fundamental aspects of ovulation are similar in *Drosophila* and mammals will accelerate the study of these and many other questions.

## Materials and Methods

### *Drosophila* genetics

Flies were reared on standard cornmeal-molasses food at 25°C unless otherwise indicated. The following Gal4 lines from the Janelia Farm collection [38] were used to label follicle cells and corpus luteum cells: R47A04 (*Oamb*), R49E12 (*5-HT2A*), R10E05 (*AstC-R2*), and R42A05 (*kay*). To knockdown *mmp1* or *mmp2* or overexpress *Timp* in adult flies, *actGal4/Cyo; tubGal80* ^*ts*^ virgin females were crossed to the following lines at 18°C and shifted to 29°C immediately after adult eclosion: *UAS-mmp1* ^*RNAi*^ (Bloomington Drosophila stock center, B31489), *UAS-mmp1* ^*RNAi2*^ [39], *UAS-mmp1* ^*RNAi3*^ (Vienna Drosophila RNAi Center, V108894), *UAS-mmp1* ^*E225A*^ (a dominant negative form of Mmp1) [40], *UAS-mmp2* ^*RNAi*^ [39], *UAS-mmp2* ^*RNAi2*^ (VDRC, V107888), *UAS-mmp2* ^*RNAi3*^ (BDSC, B31371), *UAS-Timp* [41]. To knock down *mmp1* or *mmp2* or overexpress *Timp* in follicle cells of mature egg chambers, *UAS-dcr2; R47A04* virgin females were crossed to the RNAi lines described above at 29°C. To knock down ecdysone synthesis genes, *UAS-dcr2; R47A04* virgin females were crossed *shd* ^*RNAi*^ (VDRC, V17203), *dib* ^*RNAi*^ (VDRC, V101117), or *phm* ^*RNAi*^ (VDRC, V108359). To overexpress *mmp1* or *mmp2* in mature follicle cells, *R47A04* virgin females were crossed to *UAS-mmp1* or *UAS-mmp2* [41] at 21°C. Control flies were derived from specific Gal4 driver crossed to wild-type Oregon-R. Mmp2::GFP fusion genes were generated through recombinase mediated cassette exchange of MiMIC insertion (MI02914) in the third coding intron of *mmp2* (S4 Fig.) [42]. *Mmp2-Gal4* line is from an Gal4 enhancer trap [43], and *UAS-RedStinger* (BDSC, B8547) and *UASpGFP-act79B; UAS-mCD8-GFP* were used as reporters. sqh-EYFP-Mito (BDSC, B7194) and sqh-EYFP-ER (BDSC, B7195) were used for tracking mitochondria and endoplasmic reticulum, respectively.

### Egg laying and ovulation time

Egg laying and ovulation was determined essentially as described [19]. Virgin females were aged for four to five days, and fed with wet yeast one day before experiments. To measure egg laying time (the average time between successive ovipositions), five females were mated to 10 Oregon-R males in each bottle covered with a molasses plate at 29°C and five or more bottles were set up for each genotype. The molasses plates were replaced every 22 hours and the number of eggs laid was counted for 44 hours and used to determine the average time required per egg. Egg laying time (min) was then calculated as 22 hr x 60 min / eggs laid per 22hr. To determine ovulation time, about 30 single-pair matings with one virgin female and one Oregon-R male were carried out for each genotype at 29°C for 6 hours, an interval sufficient for all females to reach a steady state level of ovulation and egg laying. Females were then frozen in -80°C for four minutes, their reproductive tracts were dissected to identify eggs inside the reproductive tract, and the percentage of females with an egg in the uterus or actively ejecting out of the uterus (U%) was calculated. Free eggs were never observed in the common oviducts in control or mutant flies (N = 196), indicating that eggs spend a negligible amount of time moving through the oviducts. In addition, we never observe more than one egg in the female reproductive tract (N = 196). Therefore, the egg laying time is partitioned into the uterus time (the average time eggs reside in the uterus or actively ejecting) and the ovulation time (the average time eggs prepare to be released from the ovary), which includes the time when the follicle protrudes into the lateral oviduct, because our study indicates that these eggs are in the process of ovulation and have not yet been released from the ovary (Fig. 2E-F, 3D, and 4E). Uterus time was then calculated as egg laying time x U%, and ovulation time = egg laying time x (1—U%). The ovulation time is a proxy for the average time needed for an egg to be released from the ovary while flies are laying eggs rapidly at steady state. The ovulation process is complex and is known to be affected by many factors, such as glandular secretions, male peptides, moisture, and egg laying substrates, whose influences are aggregated using this approach. The 95% confidence intervals were calculated correspondingly.

### Follicle cell trimming

Females were frozen in -80°C for four minutes before dissection. Ovaries were dissected out immediately afterwards, fixed with 4% EM Grade paraformadehyde, and stained with DAPI. Care was taken to make sure that two ovaries from single female were intact after staining and mounted in the same well on slides by carefully separating the ovarioles. The number of trimming follicles was scored according to the criteria that a quarter of the egg chamber at the posterior end has no follicle cells covering, and the number of mature follicles was scored according to their fully elongated dorsal appendage. The normalized trimming follicles were then calculated by the number of trimming follicles divided by the number of mature follicles in each female. For follicle cell trimming analysis in Fig. 2G, three to four days old *w* ^*1118*^ mated or unmated females were used. For analyzing trimming in females with Mmp knock down, three to four days old virgin females were mated with Oregon-R males for six hours before dissection. For trimming with misexpressing mmp1 and mmp2, follicles were directly collected from female abdominal cavity.

### Immunostaining, *in situ* zymography, and microscopy

Antibody staining followed a standard procedure [44]. Briefly, tissues were fixed in 4% EM-Grade paraformadehyde for 15 minutes and blocked in PBTG (PBS+ 0.2% Triton+ 0.5% BSA+ 2% normal goat serum). Primary antibodies were incubated overnight at 4°C and secondary antibodies were incubated for two hours at room temperature, followed by DAPI staining. The following primary antibodies were used: mouse anti-Hnt (1: 75) and anti-Arm (1:40) from Developmental Study Hybridoma Bank, rabbit anti-Shd (1:250; a gift from Michael O’Connor) and anti-Phm [25] (1:250), rabbit anti-GFP (1:4000, Invitrogen), and rabbit anti-RFP (1:2000, MBL international). Secondary antibodies were Alexa 488 goat anti-rabbit and 546 goat anti-mouse and anti-rabbit (1:1000, Invitrogen). Images were acquired using the Leica TCS SP5 confocal microscope, and assembled using Photoshop software (Adobe, Inc.). Images for yellow pigmentation of the corpus luteum were taken with the Macropod with Canon 6D camera and Olympus SZX16 stereomicroscope with Olympus DP72 color camera.

The *in situ* zymography technique for gelatinase activity was performed as previously described with minor modifications [45]. Ovaries were dissected in pre-warmed Grace’s media and incubated immediately in 100 μg/ml DQ-gelatin conjugated with fluorescein (Invitrogen) for an hour. To increase substrate penetration, the peritoneal muscle sheath was broken at the ovarian anterior. Ovaries were then fixed in 4% EM-Grade Paraformadehyde for 15 minutes and mounted for visualization.

## Supporting Information

S1 Fig Expression and function of ecdysone biosynthetic enzymes in the corpus luteum and mature follicle cells. (A-A”) Phm is detected by antibody staining in mature follicle cells (stage 14) and corpus luteum cells, but weakly in stage-12 follicle cells. Phm expression is overlapped with Endoplasmic reticulum (ER) marker. (B) Knocking down genes encoding ecdysone biosynthetic enzymes (Shd, Phm, and Dib) with R47A04-Gal4 driver causes reduction of egg laying in two days. * P<0.05, ** P<0.01.
(TIF)

S2 Fig Follicle cell trimming in paired ovaries. (A) A table showing the number of mature or trimmed follicles in each ovary of the female flies rapidly laying eggs. (B-C) shows the two and only two trimmed follicles (outlined) from two ovaries of the same fly. The trimmed follicle in (B) protruded into the oviduct (arrow) and lost more follicle cell covering at their posterior end than the one in (C).
(TIF)

S3 Fig Two-day egg laying of females with reduced Mmp activity in adult. (TIF)

S4 Fig Generation and verification of Mmp2::GFP fusion protein. (A) Schematic diagram of Mmp2 genomic locus, MI02914 insertion site, and Mmp2::GFP exchange cassette. (B) Recombinase-mediated cassette exchange of MI02914 and PCR verification of correct insertion of mmp2::GFP fusion product. (C-D) Mmp2::GFP1 is the correct insertion and shows GFP expression in corpus luteum cells (outlined) and the posterior follicle cells of stage 14 egg chamber (arrow) but not stage 12 egg chamber. Mmp2::GFP2 is the incorrect insertion and does not show GFP expression.
(TIF)

S5 Fig Expression of *Mmp2-Gal4*, *47A04-Gal4*, *and 42A05-Gal4*. Gal4 expression was indicated by a *UAS-mCD8-GFP* reporter. (A-C) *Mmp2-Gal4* is expressed in both anterior and posterior follicle cells of a mature follicle. (B) and (C) is the enlarged areas in (A). (D) *Mmp2-Gal4* is expressed in the anterior and posterior cells in corpus luteum (outlined). (E-F) *47A04-Gal4* is expressed in mature follicle cells (E) and the posterior end of the oviduct cells, but not in neurons innervating the reproductive tract (F). Oviduct is outlined in F. (G-H) *42A05-Gal4* is expressed in anterior (arrow) and posterior tip (arrowhead) follicle cells of mature egg chambers (G) and some interstial cells in seminal receptacle (SR), but it is not expressed in the oviduct or uterus (Ut) or neurons innervating the reproductive tract. High auto-fluorescence was detected in the egg reside in the uterus. Oo: Oocyte.
(TIF)
