# Supplementary figures and images for: Matrix Metalloproteinase 2 Is Required for Ovulation and Corpus Luteum Formation in Drosophila
Source: PLoS Genet. 2015 Feb 19;11(2):e1004989. doi: 10.1371/journal.pgen.1004989 (PMC4335033; doi:10.1371/journal.pgen.1004989)

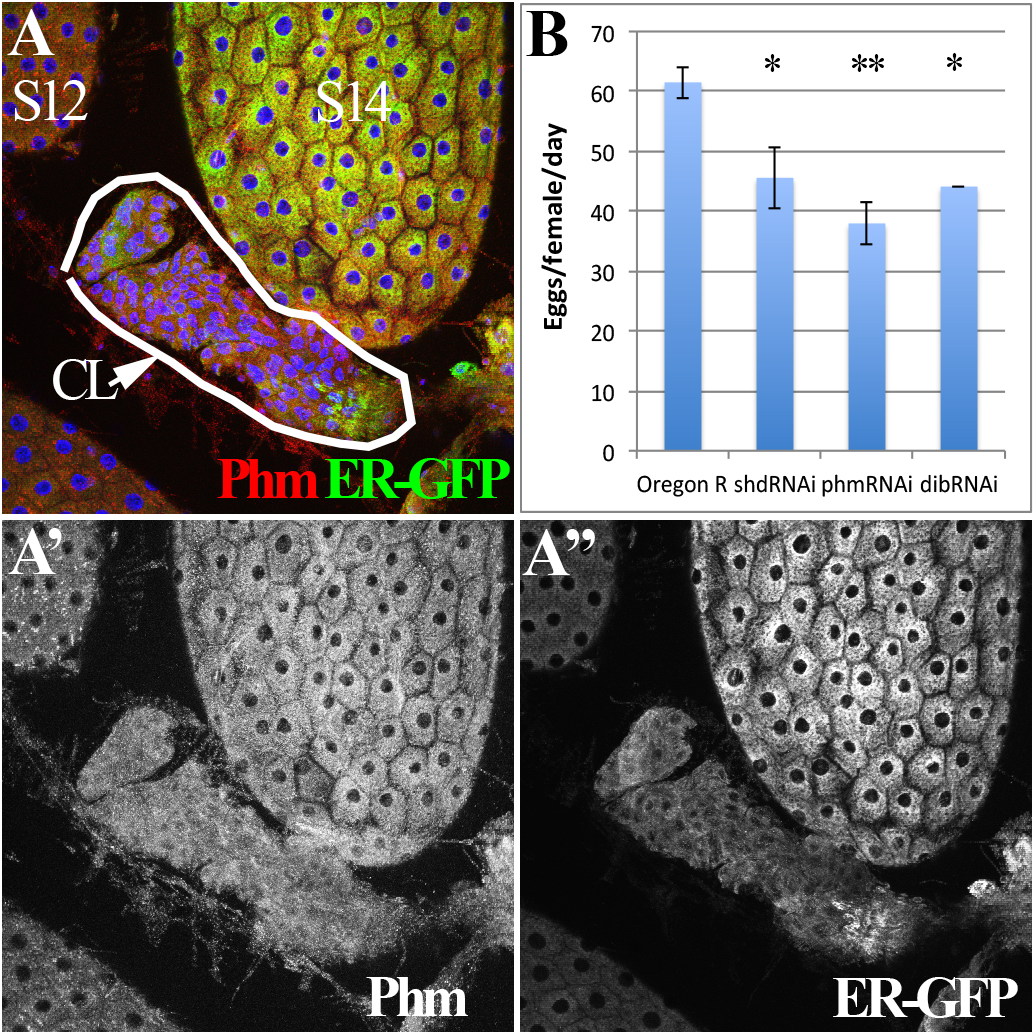

Supplement: S1 Fig — (A-A”) Phm is detected by antibody staining in mature follicle cells (stage 14) and corpus luteum cells, but weakly in stage-12 follicle cells. Phm expression is overlapped with Endoplasmic reticulum (ER) marker. (B) Knocking down genes encoding ecdysone biosynthetic enzymes (Shd, Phm, and Dib) with R47A04-Gal4 driver causes reduction of egg laying in two days. * P<0.05, ** P<0.01. (TIF) [file pgen.1004989.s001.tif]

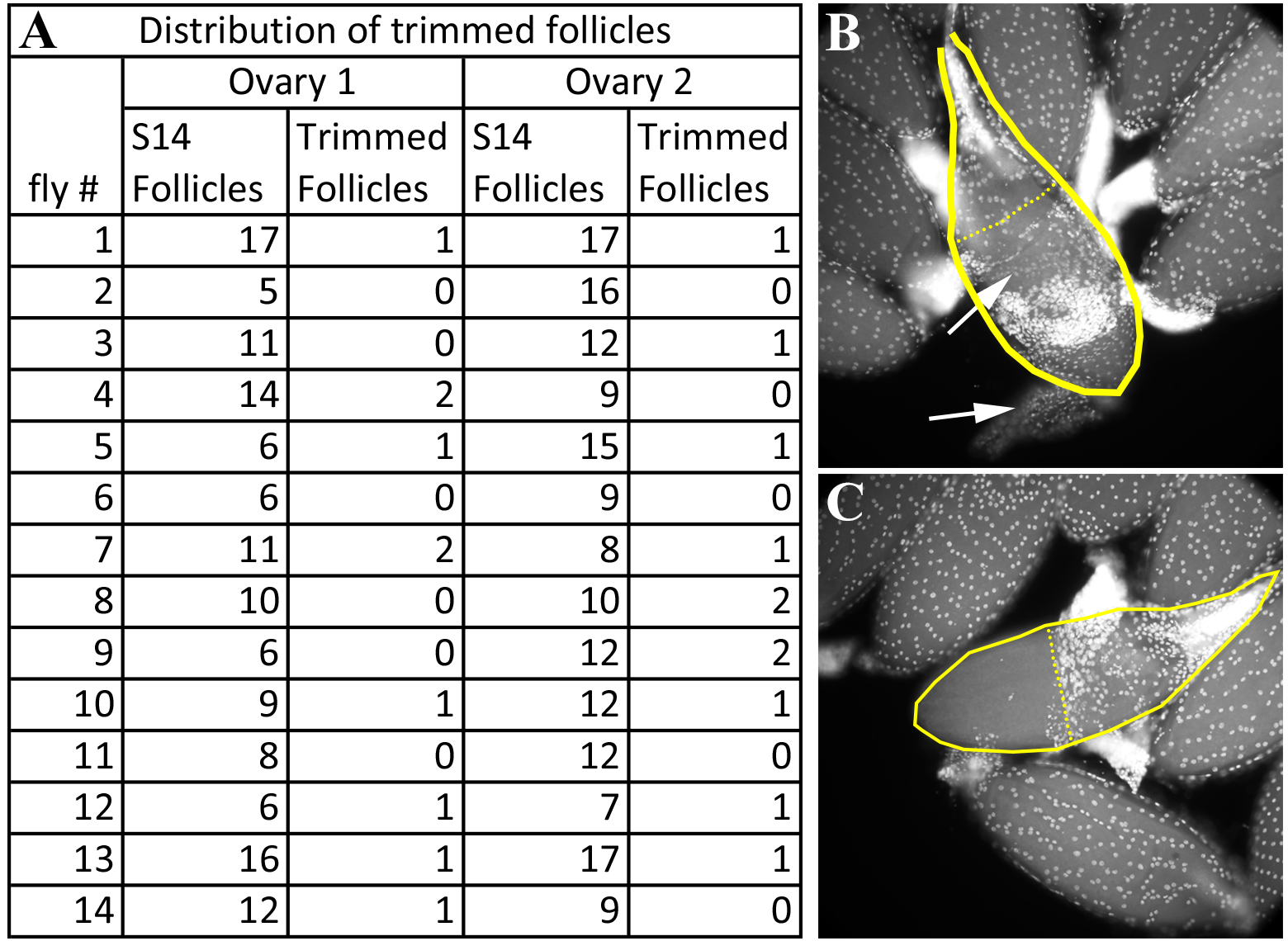

Supplement: S2 Fig — (A) A table showing the number of mature or trimmed follicles in each ovary of the female flies rapidly laying eggs. (B-C) shows the two and only two trimmed follicles (outlined) from two ovaries of the same fly. The trimmed follicle in (B) protruded into the oviduct (arrow) and lost more follicle cell covering at their posterior end than the one in (C). (TIF) [file pgen.1004989.s002.tif]

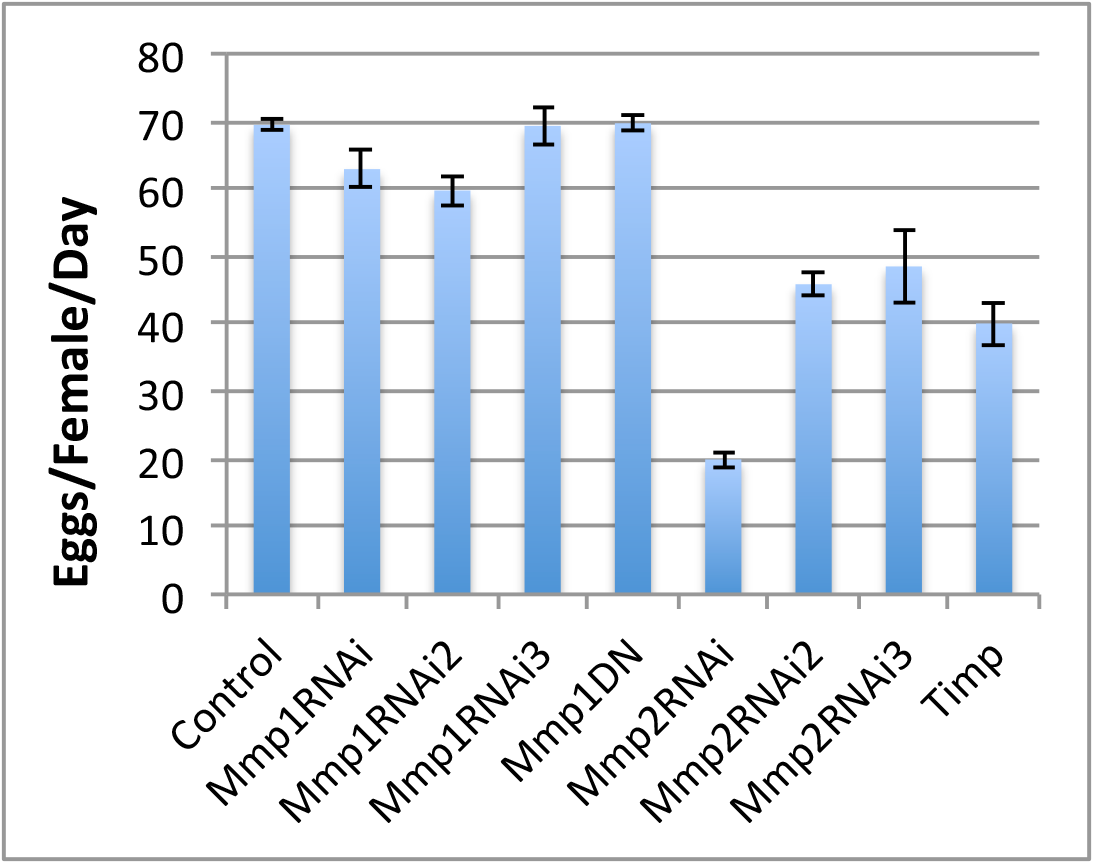

Supplement: S3 Fig — (TIF) [file pgen.1004989.s003.tif]

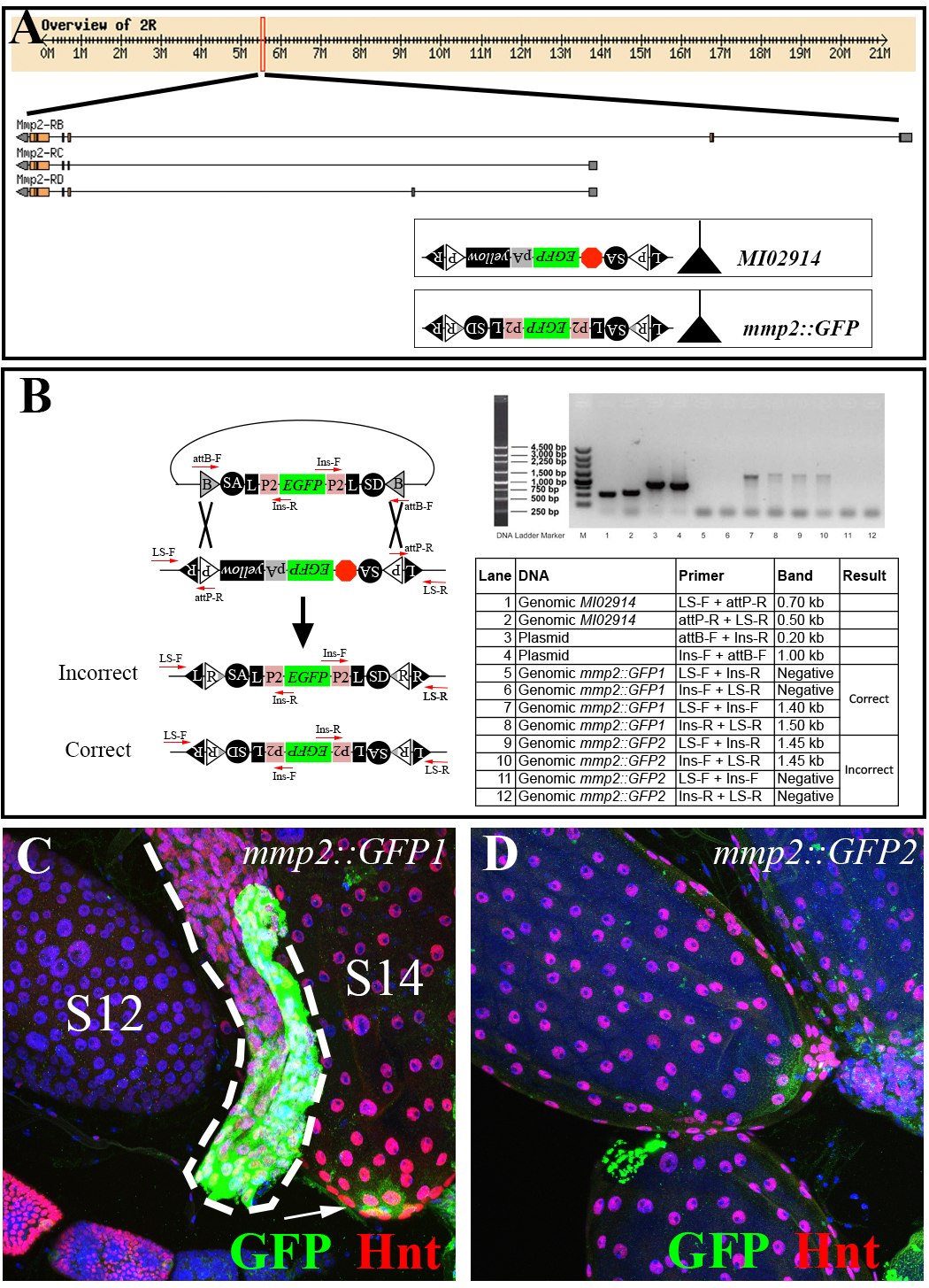

Supplement: S4 Fig — (A) Schematic diagram of Mmp2 genomic locus, MI02914 insertion site, and Mmp2::GFP exchange cassette. (B) Recombinase-mediated cassette exchange of MI02914 and PCR verification of correct insertion of mmp2::GFP fusion product. (C-D) Mmp2::GFP1 is the correct insertion and shows GFP expression in corpus luteum cells (outlined) and the posterior follicle cells of stage 14 egg chamber (arrow) but not stage 12 egg chamber. Mmp2::GFP2 is the incorrect insertion and does not show GFP expression. (TIF) [file pgen.1004989.s004.tif]

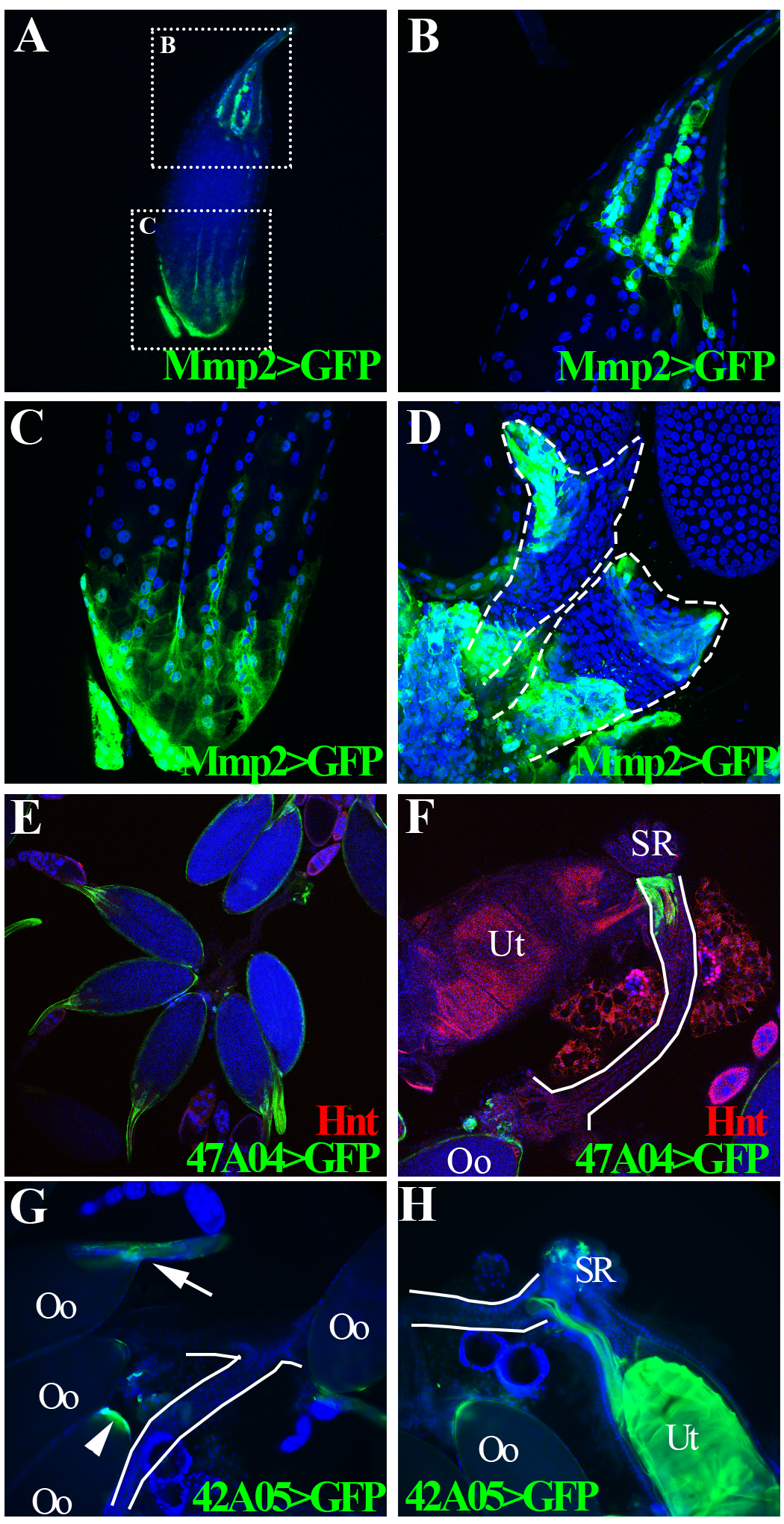

Supplement: S5 Fig — Gal4 expression was indicated by a UAS-mCD8-GFP reporter. (A-C) Mmp2-Gal4 is expressed in both anterior and posterior follicle cells of a mature follicle. (B) and (C) is the enlarged areas in (A). (D) Mmp2-Gal4 is expressed in the anterior and posterior cells in corpus luteum (outlined). (E-F) 47A04-Gal4 is expressed in mature follicle cells (E) and the posterior end of the oviduct cells, but not in neurons innervating the reproductive tract (F). Oviduct is outlined in F. (G-H) 42A05-Gal4 is expressed in anterior (arrow) and posterior tip (arrowhead) follicle cells of mature egg chambers (G) and some interstial cells in seminal receptacle (SR), but it is not expressed in the oviduct or uterus (Ut) or neurons innervating the reproductive tract. High auto-fluorescence was detected in the egg reside in the uterus. Oo: Oocyte. (TIF) [file pgen.1004989.s005.tif]
